# Editorial: Big Data Analytics for Precision Health and Prevention

**DOI:** 10.3389/fdata.2021.835353

**Published:** 2022-01-12

**Authors:** Enrico Capobianco, Jun Deng

**Affiliations:** ^1^Institute of Data Science and Computing, University of Miami, Coral Gables, FL, United States; ^2^Department of Chemical Sciences and Materials Technologies, Institute of Organic Synthesis and Photoreactivity, National Research Council of Italy (CNR), Bologna, Italy; ^3^Department of Therapeutic Radiology, Yale University School of Medicine, New Haven, CT, United States

**Keywords:** precision health, data science, machine learning, artificial intelligence, medical decision analysis

In the oncological context, big data analyses are quite complex due to heterogeneous data structures and varieties (Agarwala et al., 2018). The defining characteristic of precision health is the possibility to translate a wealth of science results into clinical practice and allow to target more personalized therapies, interventions, and prevention in general care (Krzyszczyk et al., 2018). Two factors are contributing to the success of such tasks: (a) increased volumes and types of available biomedical data offering potentially disruptive insights on cancer (and other diseases) (Bi et al., 2019; Krassowski et al., 2020); (b) focus on assimilation of the diversity that data brings and its integration in unifying modeling approaches (Parikh et al., 2019; Bekisz and Geris, 2020; Cappelli et al., 2020; Niida et al., 2020). Combining health data requires suitable harmonization of analytical tools, thus the need of developing value-seeking solutions. This Research Topic has included a few novel developments on big data analytics for precision health and prevention. Below, a list of contributed topics is briefly described with emphasis on the main results that were obtained.

“*Artificial Intelligence Based Approaches to Identify Molecular Determinants of Exceptional Health and Life Span-An Interdisciplinary Workshop at the National Institute on Aging*,” by Moore et al. describes how the AI's potential may directly or indirectly identify factors explaining human exceptional health and life span remains largely unexplored, as wells as the expected translation into data- and evidence-driven interventions. Revealing such conditions through novel AI approaches defines the physiological complexity that governs health and life span and guides new therapeutic developments for healthy aging. A National Institute on Aging (NIA) workshop held in August 2018 is used as a reference in this work for indicating emerging AI applications in the above contexts.

In “*Robust Machine Learning for Colorectal Cancer Risk Prediction and Stratification*,” by Nartowt et al. the focus is on mass screening for colorectal cancer (CRC), third cancer in prevalence and mortality in US. Seven supervised machine learning algorithms were evaluated through models trained and tested with the National Health Interview Survey (NHIS) and the Prostate, Lung, Colorectal, Ovarian Cancer Screening (PLCO) datasets. Missing data were treated by several imputation methods and the one with expectation-maximization imputation was the best with a concordance of 0.70 ± 0.02, sensitivity of 0.63 ± 0.06, and specificity of 0.82 ± 0.04. Risk stratification for CRC in the NHIS and PLCO datasets showed very low misclassification rates, i.e., 2% of negative cases (found with high risk) and 6% of positive cases (found with low risk). As modeling the CRC-free probability with Kaplan-Meier estimators identified low-, medium-, and high CRC-risk groups with statistically significant separation, the authors concluded that the trained artificial neural network represents an effective screening tool for early intervention and prevention.

In “*Reducing Annotation Burden Through Multimodal Learning*,” by Lopez et al. deep learning is studied for the aim of multimodal data fusion, i.e., classifying radiological images and text reports. Early, late and model fusion techniques were considered as prototypes and classification performance was comparatively assessed between multimodal and unimodal learning models in order to find how many labeled data can make the two classifications of comparable value. It is shown that multimodal fusion can achieve similar performance with less labeled (training) data, especially with early techniques. This result directly translates into less annotation burden for domain experts.

“*PECLIDES Neuro: A Personalisable Clinical Decision Support System for Neurological Diseases*,” by Müller and Lio, is about the ability of machine learning techniques to be transparent by responding to criteria of explainability of the methods they use for diagnostic purposes. The proposed algorithm, which is named “Personalisable Clinical Decision Support System” (PECLIDES), provides insights into the decision-making diagnostic process. Based on random forests, PECLIDES presents a rule set open to the physician's observation, who can thus shape it when assessing intra-disease heterogeneity or inter-disease comparisons. Although applicable to various decision settings, the work-s focus is on neurological diseases (PECLIDES Neuro).

Bikia et al. presented “*AI-Based Estimation of End-Systolic Elastance From Arm-Pressure and Systolic Time Intervals”* with reference to the study of left ventricular end-systolic elastance (E_es_), a major determinant of cardiac systolic function and ventricular-arterial interaction. Accurate E_es_ estimation requires the use of the echocardiographic ejection fraction (EF) to inform on the stroke volume as a fraction of end-diastolic volume (EDV) to be combined with its measurement. The study introduces a novel artificial intelligence approach to estimate E_es_ by using the information embedded in clinically relevant systolic time intervals, the pre-ejection period (PEP) and the ejection time (ET). With virtual subjects (*n* = 4,645) from a previously validated *in-silico* model, the Extreme Gradient Boosting regressor was employed to model E_es_ using as inputs arm cuff pressure, PEP, and ET, and obtaining prediction of E_es_ with normalized RMSE equal to 9.15% (*r* = 0.92) for a wide range of E_es_ values from 1.2 to 4.5 mmHg/ml. Notably, the model was found less sensitive to measurement errors (±10–30% of the actual value) in blood pressure and highly sensitive measurements errors in the systolic timing features, but overall offers a clinically applicable method for assessing left ventricular systolic function.

While only covering a few latest developments, this Research Topic reveals a tip of the iceberg on big data analytics' usefulness for precision health and prevention. With increased and qualitatively valid collaborations between human intelligence and artificial intelligence, it is anticipated that more widespread applications of big data analytics in individuals' health could lead to earlier detection of diseases, more precise treatments and more effective interventions, which would significantly improve the health care and quality of life for millions of people in the years to come.

## Author Contributions

EC wrote the manuscript. JD reviewed the manuscript. All authors contributed to the article and approved the submitted version.

## Funding

JD was supported by the National Institute of Biomedical Imaging and Bioengineering of the National Institutes of Health under Award Number R01EB022589, by the National Science Foundation under Award Number DMS 1918925, by the National Cancer Institute under Award Number 21X130F, and by the Department of Energy under Award Number DE-SC0021655 to JD. EC was supported by the Grant NSF 19-500 (Award No. DMS 1918925/1922843; Years 2019–2022).

## Author Disclaimer

The content of this work is solely the responsibility of the authors and does not necessarily represent the official views of the listed institutions.

## Conflict of Interest

The authors declare that the research was conducted in the absence of any commercial or financial relationships that could be construed as a potential conflict of interest.

## Publisher's Note

All claims expressed in this article are solely those of the authors and do not necessarily represent those of their affiliated organizations, or those of the publisher, the editors and the reviewers. Any product that may be evaluated in this article, or claim that may be made by its manufacturer, is not guaranteed or endorsed by the publisher.

## References

[B1] Agarwala V. Khozin S. Singal G. O'Connell C. Kuk D. Li G.. (2018). Real-world evidence in support of precision medicine: clinico-genomic cancer data as a case study. Health Aff. 37, 765–772. 10.1377/hlthaff.2017.1579 29733723

[B2] Bekisz S. Geris L. (2020). Cancer modeling: from mechanistic to data-driven approaches, and from fundamental insights to clinical applications. J. Comput. Sci. 46:101198. 10.1016/j.jocs.2020.101198

[B3] Bi W. L. Hosny A. Schabath M. B. Giger M. L. Birkbak N. J. Mehrtash A.. (2019). Artificial intelligence in cancer imaging: clinical challenges and applications. CA Cancer J. Clin. 69, 127–157. 10.3322/caac.21552 30720861 PMC6403009

[B4] Cappelli E. Cumbo F. Bernasconi A. Canakoglu A. Ceri S. Masseroli M.. (2020). OpenGDC: unifying, modeling, integrating cancer genomic data and clinical metadata. Appl. Sci. 10:6367. 10.3390/app10186367

[B5] Krassowski M. Das V. Sahu S. K. Misra B. B. (2020). State of the field in multi-omics research: from computational needs to data mining and sharing. Front. Genet. 11:1598. 10.3389/fgene.2020.610798 33362867 PMC7758509

[B6] Krzyszczyk P. Acevedo A. Davidoff E. J. Timmins L. M. Marrero-Berrios I. Patel M.. (2018). The growing role of precision and personalized medicine for cancer treatment. Technology 6, 79–100. 10.1142/S2339547818300020 30713991 PMC6352312

[B7] Niida A. Hasegawa T. Innan H. Shibata T. Mimori K. Miyano S. (2020). A unified simulation model for understanding the diversity of cancer evolution. Peer J. 8:e8842. 10.7717/peerj.8842 32296600 PMC7150545

[B8] Parikh R. B. Gdowski A. Patt D. A. Hertler A. Mermel C. Bekelman J. E. (2019). Using big data and predictive analytics to determine patient risk in oncology. Am. Soc. Clin. Oncol. Educ. Book 39, e53–8. 10.1200/EDBK_238891 31099672

